# Cognitive dissonance resolution depends on episodic memory

**DOI:** 10.1038/srep41320

**Published:** 2017-01-23

**Authors:** Mariam Chammat, Imen El Karoui, Sébastien Allali, Joshua Hagège, Katia Lehongre, Dominique Hasboun, Michel Baulac, Stéphane Epelbaum, Agnès Michon, Bruno Dubois, Vincent Navarro, Moti Salti, Lionel Naccache

**Affiliations:** 1 INSERM, U 1127, F-75013, Paris, France; 2 Institut du Cerveau et de la Moelle épinière, ICM, PICNIC Lab, F-75013, Paris, France; 3 CENIR, Centre de NeuroImagerie de Recherche, Paris, France; 4 Sorbonne Universités, UPMC Univ Paris 06, Faculté de Médecine Pitié-Salpêtrière, Paris, France; 5 AP-HP, Groupe hospitalier Pitié-Salpêtrière, Department of Neurology, Paris, France; 6 AP-HP, Groupe hospitalier Pitié-Salpêtrière, Department of Neurophysiology, Paris, France; 7 Ben-Gurion University, Department of Brain and Cognitive Science, Beer-Sheva, Israel

## Abstract

The notion that past choices affect preferences is one of the most influential concepts of social psychology since its first report in the 50 s, and its theorization within the cognitive dissonance framework. In the free-choice paradigm (FCP) after choosing between two similarly rated items, subjects reevaluate chosen items as more attractive and rejected items as less attractive. However the relations prevailing between episodic memory and choice-induced preference change (CIPC) remain highly debated: is this phenomenon dependent or independent from memory of past choices? We solve this theoretical debate by demonstrating that CIPC occurs exclusively for items which were correctly remembered as chosen or rejected during the choice stage. We used a combination of fMRI and intra-cranial electrophysiological recordings to reveal a modulation of left hippocampus activity, a hub of episodic memory retrieval, immediately before the occurrence of CIPC during item reevaluation. Finally, we show that contrarily to a previous influential report flawed by a statistical artifact, this phenomenon is absent in amnesic patients for forgotten items. These results demonstrate the dependence of cognitive dissonance on conscious episodic memory. This link between current preferences and previous choices suggests a homeostatic function of this regulative process, aiming at preserving subjective coherence.

According to the cognitive dissonance theory initially framed in the 50 s^1^ by Festinger, our past choices influence our current preferences. Since then, several experimental paradigms have been designed to test this theory including the free-choice paradigm (FCP)^2^. However, in spite of a rich literature the relations prevailing between episodic memory and choice-induced preference change (CIPC) remain highly debated: is CIPC dependent^3^ or independent^4^,^5^ from memory of past choices? In the present work we report a series of experiments using the FCP to solve this long-lasting issue.

A typical FCP experiment is composed of three blocks. First, subjects rank or rate items according to their desirability (e.g.: food items, car models, holiday destinations, etc.). Second, they are engaged in a forced-choice task during which they have to choose between two closely rated items. Finally, they perform a second rating on the same items. Choice-induced preference change is defined by a tendency to increase ratings of chosen items, and to decrease those of rejected items. This ‘spreading of alternatives’, or ‘spread’, is the hallmark of this phenomenon, and has been considered as diagnostic of CIPC.

Since 2001 a cumulative series of empirical studies progressively introduced the notion that CIPC was independent from conscious episodic memory, and from the activity of hippocampal structures. A significant spreading of alternatives was observed in amnesic patients suffering from severe anterograde amnesia due to hippocampal lesion or dysfunction, as well as in normal controls under conditions of high cognitive load^4^ or who forgot their choices for odorant items^6^. Similarly, spread was reported after a year-long delay period between choice and second rating^5^, as well as in Capucin monkeys and in young infants^7^,^8^. Moreover, this encapsulated and automatic unconscious updating of values was associated with activity changes within the ‘value-network’ including caudate nuclei and orbito-frontal cortices^9^,^10^.

However a major statistical artifact was identified by Chen and Risen^11^ who showed that spreading of alternatives could be observed in the absence of genuine preference change. Their claim relied on two assumptions. First, they presumed that ratings are noisy measures of subjects’ preferences. Second they suggested that subjects’ choices give additional information about their preferences. Accordingly, if two items, A and B, were similarly rated by a subject, and he chooses A over B, then the most probable account would be that item A is actually preferred over item B, and that initial identical ratings corresponded respectively to under-estimation and over estimation of genuine preferences for A and B. Therefore, as a result of regression to the mean, one could expect an increase of rating for item A and a decrease of rating for item B during the second rating, without any change in preferences for these items. A univocal way to control for this artifact consists in using a new control condition during which subjects perform their two ratings before the choice stage (RRC), in addition to the traditional rating/choice/rating sequence (RCR).

Since this work, which reveals a severe caveat that may undermine many if not all previous reports, a few studies reported genuine CIPC but without controlling for recall of previous choices in episodic memory (see ref. 3 for a recent review and discussion).

Crucially, we recently reported in normal controls that CIPC was strongly associated with episodic memory: spreading of alternatives was significant in the critical RCR condition, and larger than in the RRC control condition, only for items whose choices were correctly remembered in a final post-experimental block^3^. As we were interested in testing whether subjects remembered the episodes of their choices, we considered items as remembered only if subjects correctly reported whether they had chosen or rejected each of the two coupled items. This decision aimed at avoiding the memory test to reflect a new choice rather than a memory task: if the subject sees an item that he likes he will tend to answer “I chose it” even without checking into memory. Correct answers to a pair of items rated similarly during R1 increases the probability that the subject relied on episodic memory during the memory test.

This strong association is suggestive of a causal relation between CIPC and episodic memory. In the present work we strengthen the plausibility of this causal hypothesis. We replicate our previous behavioral findings and reveal the left hippocampus as a possible key player of such a possible causal mechanism.

In order to establish a direct link between CIPC and episodic memory of previous choices we aimed at measuring neural activity within episodic memory networks precisely during the second rating session which is the moment where CIPC is detected in the behavior. Indeed, our behavioral paradigm (see Fig. 1 and online supplementary information (SI) for Materials & Methods) probes memory of choices only at the end of the experiment, making it difficult to test whether subjects were actually retrieving their choices when performing the second rating.

## Results

We recruited a group of 20 control subjects. Behavioral data strictly replicated our previous results^3^ (see Fig. 2). A main effect of memory was found (F(1,943) = 51.87; p < 10^−11^), as well as a main effect of condition(F(1,980) = 6.71; p = 0.009). Crucially, the critical and predicted interaction between memory and condition was significant (F(1,992) = 11.14; p < 0.001): while no difference of spreads was observed between RCR and RRC conditions for forgotten items (t(19) = −0.57; p = 0.6), the spread was significantly larger in the RCR condition than in the RRC condition for remembered items (t(19) = 3.11; p = 0.005). These subjects’ memory performance was at 45.7% (±13.5) correctly remembered items.

We then turned to fMRI analyses and first verified that our task engaged the brain value system^12^,^13^,^14^ by identifying regions whose activity correlated with subjects’ ratings (in Rating 1 session, see SI). A significant positive correlations between whole-brain fMRI signal and preference ratings was observed within the striatum (mainly the anterior part of the caudate nucleus bilaterally) and all its major inputs including notably the left ventro-medial prefrontal cortex, the anterior and posterior cingulate cortices and the precuneus (see Fig. 2b and Table S1). This result replicates the typical pattern of brain valuation system activation previously reported in the literature^15^,^16^,^17^. Next, we tested whether the memory effects collected at the end of the experiment could be correlated with relevant brain activations during the second rating session. Given our strong prediction about hippocampus implication in CIPC, we first performed a region of interest analysis within bilateral hippocampi cortices (using the WFU PickAtlas toolbox for SPM). In close parallel to behavior, BOLD signal showed the predicted critical interaction between memory and condition (see Fig. 2c) which is the hallmark of genuine CIPC. Post-hoc tests confirmed that fMRI signal was larger for RCR than for RRC items, but exclusively for remembered items and not for forgotten ones. Note that the same analysis computed on fMRI signal recorded during rating 1 did not show any significant effect. We then ran a whole brain analysis in which we probed brain regions recorded during Rating 2 showing the crucial interaction between condition (RCR > RRC) and memory (Remembered > Forgotten). Crucially, only the left hippocampus (peak coordinates = [−14 −24 −12]) showed such an effect (p < 0.001 uncorrected in a minimum of 40 contiguous voxels).

**Figure 1:**
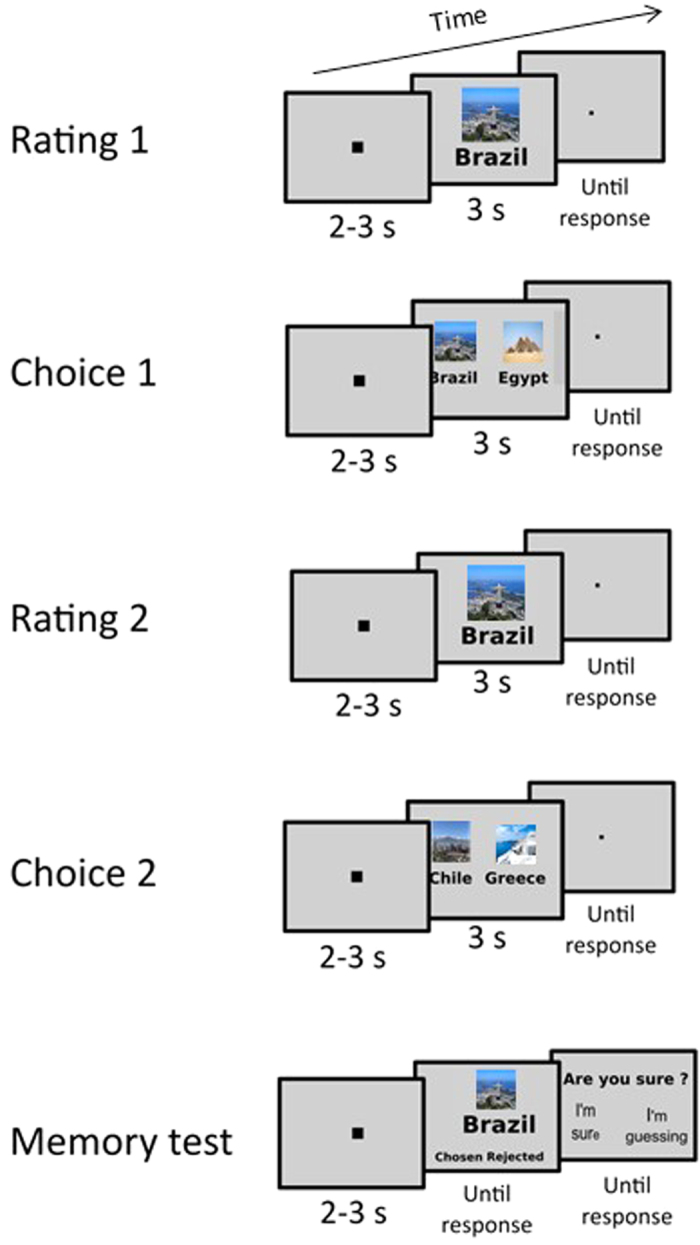
Experimental Design. The experimental paradigm common to all experiments included 5 steps. First, subjects were asked to rate all items on an eight points scale. Then they chose between similarly rated pairs of items (only half of items from Rating 1 were presented in this block). In the third block they were asked to rate all items again. In the fourth block, subjects had to choose between the second half of items. Finally, they were presented with a memory test, which assessed both their objective (forced-choice task) and subjective memory. Slight differences distinguished the purely behavioral experiments from the fMRI and SEEG experiments as detailed in the procedure (see SI). The four images displayed in this figure are free of use for commercial usage (Rio de Janeiro Corcovado mountain by Artyominc https://commons.wikimedia.org/wiki/File:Christ_on_Corcovado_mountain.JPG (CC BY-SA 3.0 https://creativecommons.org/licenses/by-sa/3.0/deed.en); Gizah pyramids by Ricardo Liberato https://en.wikipedia.org/wiki/File:All_Gizah_Pyramids.jpg (CC BY-SA 2.0 https://creativecommons.org/licenses/by-sa/2.0/deed.en); Santiago Chile by Patrick Coe https://commons.wikimedia.org/wiki/File:Santiago_Chile.jpg (CC BY 2.0 https://creativecommons.org/licenses/by/2.0/deed.en); Greek landscape https://pixabay.com/fr/gr%C3%A8ce-mer-vue-sur-la-mer-sud-905559/ is in the public domain with no attribution required (CC0 https://creativecommons.org/publicdomain/zero/1.0/deed.fr).

**Figure 2:**
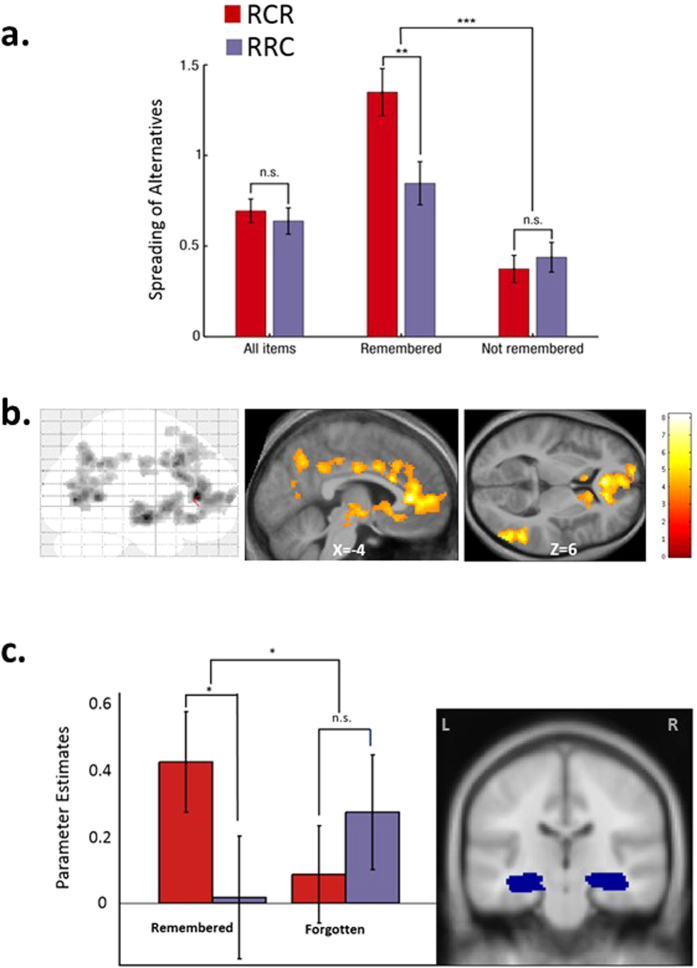
fMRI experiment revealed the contribution of hippocampus to CIPC during the second rating. (**a**) Behavior: while no CIPC was observed when analyzing all items (left bars), the critical interaction between condition (RCR/RRC) and memory (Remembered/Forgotten) that is the hallmark of CIPC was found highly significant in the predicted direction (right bars). Post-hoc contrasts confirmed that CIPC was present exclusively for remembered items. (‘***’ for p < 0.001; ‘**’ for 0.001 ≤ p < 0.01; ‘*’ for 0.01 ≤ p < 0.05; ‘n.s.’ for non-significant). (**b**) fMRI BOLD signal correlated with ratings within the classical brain valuation system including the caudate nucleus bilaterally, the left ventro-medial prefrontal cortex, the anterior and posterior cingulate cortices and the precuneus (p < 0.001 for height (uncorrected) and p < 0.05 Family wise error (FWE) cluster correction). (**c**) Paralleling subjects’ behavior, the interaction between condition and memory was found to be significant in the BOLD signal during the second rating within the region of interest defined by the two hippocampi.

**Figure 3:**
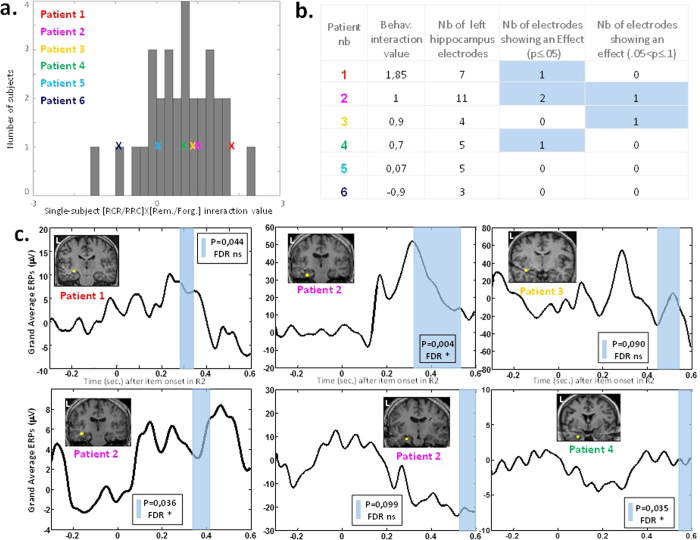
SEEG experiment revealed the contribution of hippocampus to CIPC before the behavioral response during the second rating. (**a**) Patients’ individual interaction values belonged to the distribution of interactions observed in the normal population (N = 30 controls)^19^. (**b**) Table summarizing behavioral and SEEG results of each SEEG patient. Note that 6 out of 35 electrodes with a significant critical interaction, and that these effects were observed in each of the 4 patients showing a positive CIPC in their behavior (patients 1 to 4). (**c**) Paralleling behavior and fMRI, the interaction between condition and memory during rating 2 session was observed in contacts located in the vicinity (≤30 mm) of the fMRI peak of activation within the left hippocampus. Electrodes from 4 patients showed the predicted modulation of activity within temporal window (200–600 ms). For each electrode P-value computed with a non-parametric Monte-Carlo based permutation statistics is indicated for each electrode, as well its significance to multiple testing correction using a FDR procedure (‘*’ for significant; ‘ns’ for non-significant).

Having validated the implication of the left hippocampus during the second rating we completed this result with a time-resolved technic, in order to check whether this region was modulated during the time-window associated with episodic memory retrieval (200–600 ms)^18^ and distant from the response-time window (see Fig. 1). To do so, we recorded 6 epileptic patients consecutively implanted in this region while they performed our task, and then analyzed their intracranial electrophysiological recordings in the vicinity (≤30 mm) of the left hippocampus fMRI peak of activation. Individual mean RTs ranged from 756–2182 ms (mean ± sd = 1142 ± 533 ms). Their memory performance was at 31.6% (±10.5) correct answer. For each patient, we first compared their behavioral interaction (RCR/RRC X Remembered/Forgotten) with those observed in controls^19^. All patients belonged to the distribution observed in controls, and their individual interaction value ranged from −0.9 to 1.85 (see Fig. 3a and b and Table S1). We then analyzed intra-cerebral event-related potentials (ERPs) within the 200–600 ms temporal window following item presentation during Rating 2 session, and tested the critical interaction between condition (RCR/RRC) and memory (Remembered/Forgotten) (see Fig. 3b and c). Non-parametric Monte Carlo based permutation statistics allowed us to identify 6 out of 35 electrodes with a significant or close to significance critical interaction. Importantly we could show, at the group level, that the presence of an ERP effect was associated with the critical behavioral interaction: while an ERP effect was observed in the 4 patients showing a positive spread (individual interaction value ≥0.57 that is the mean value of this interaction within the group of fMRI normal controls), no effect could be detected in the other 2 patients with negative or null spread (see Fig. 3b). Bayesian testing of the contingency table crossing the presence/absence of the critical interaction in behavioral and ERP data revealed strong evidence in favor of a link between behavioral and ERP signatures of CIPC (one-sided BF10 = 13.7; see SI for details).

These electrophysiological results confirm the fMRI findings, and further demonstrate that left hippocampus activity is modulated during rating 2 session according to the behavioral hallmark of CIPC, and in agreement with the episodic memory measure collected at the end of experiment. This new result strengthens our hypothesis of a causal link between episodic memory and CIPC.

**Figure 4:**
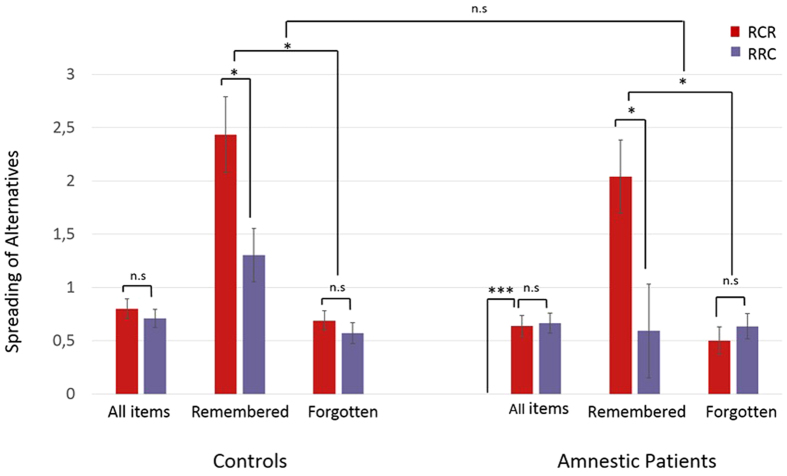
Amnesic patients with hippocampal dysfunction show CIPC exclusively for remembered items. The triple interaction between group (matched controls/aMCI), condition (RCR/RRC) and memory (remembered/forgotten) was not significant. Post-hoc analysis confirmed that while patients forgot more, they showed the very same pattern of behavioral results: CIPC was found significant exclusively for remembered items. We could also replicate the regression to the mean statistical artifact (RCR all items versus zero on the red leftmost bar of patients) present in the Lieberman *et al*.^4^ study. No CIPC was found in the absence of explicit memory of previous choices.

At this stage, however, one influential empirical result may still suggest that episodic memory does not play a causal role in CIPC. Indeed, in 2001 Lieberman and colleagues reported a significant CIPC for forgotten items in amnesic patients with hippocampal damage^4^. In the light of our results this finding could suggest that memory is simply correlated with CIPC in non-amnesic controls, through a confound between memory and attentional allocation during choice stage: the most attended items being the most prone to be remembered. In sharp contrast, and according to our hypothesis, we considered that the result of Lieberman *et al*., obtained before the discovery of Chen and Risen, should be invalidated after controlling properly for the regression to the mean artifact. Our precise prediction was that these patients who forget more, should still show the very same interaction between memory and condition: CIPC should be observed only for remembered items. In order to test this last prediction, we recruited a group of 12 patients suffering from an amnesic mild cognitive impairment (aMCI) of neurodegenerative origin associated with a moderate hippocampus atrophy. A detailed neuropsychological battery of tests (including the Mini-Mental Status Examination (MMSE)^20^, the Grober and Buschke test of verbal episodic memory^21^ and the Frontal Assessment Battery^22^) confirmed the relatively pure amnesic syndrome affecting memory consolidation related to hippocampal activity, in contrast with preservation of executive and instrumental functions (see Table S3). These patients performed our FCP ‘Holidays destination’ task (see Fig. 1), and were compared to a control group matched for sex, age and education (see Table S4). As expected, patients had more memory impairments than matched controls. On average, patients correctly remembered only 26.8% (±7.6) of their choices, while matched controls performed better with 36.3% (±10.7) correct answers (t(23) = −2.32, p = 0.04). We then analyzed spreads within an ANOVA crossing group (patients/controls) with condition (RCR/RRC) and memory (Remembered/Forgotten). A main effect of memory was found (F(1,1426.75) = 69.43; p = 10^−15^)) with a larger spread for remembered pairs. Most importantly an interaction was observed between memory and sequence (F(1,1421.19) = 5.76 p = 0.01)). This interaction did not interact with group factor (F(1,1421.19) = 0.02; p = 0.88)). Post-hoc tests confirmed that the difference between RCR and RRC was significant exclusively for remembered items t(23) = 2.1 p = 0.04, and not for forgotten items t(23) = −0.3 p = 0.71. In other terms, amnesic patients forget more than controls, but show CIPC only for remembered items, as normal controls do. No evidence of CIPC was observed for forgotten items, neither in controls, nor in amnesic patients. However, given that the objective performance criterion we used to categorize choices as remembered or forgotten differed only weakly from chance-level in patients (25%, unilateral p-value of z-test = 0.1), one may hypothesize that remembered items may have actually included some forgotten items in patients, and thus that a CIPC could still be observed in the absence of episodic memory. We therefore used a more restrictive criterion by requiring a choice to be considered as remembered only if both objective and subjective memory performance were correct (see Materials & Methods). This new analysis confirmed our prediction by showing a significant interaction between condition and memory both in patients (F(1,712.41) = 7.78; p = 0.005) and in controls (F(1,713.9) = 3.71; p = 0.05), without interaction between groups (F(1,1426.22) = 1.09; p = 0.30; see Fig. 4). Crucially, this more restrictive analysis confirmed that, in patients, no difference of spread was observed between RCR and RRC conditions for forgotten items (t(11) = −0.7, p = 0.47). In order to strengthen the value of this negative result, we computed the Bayesian Factor supporting H0 (BF_01_ = 4.4) which indicated a positive evidence in favor of H0. Similarly to Lieberman *et al*. we observed a massive spread in the critical RCR condition (t(11) = 6.2, p < 10^−4^). However, our experiment demonstrates that this spread is not larger than the one observed in the RRC condition. In other terms our behavioral results obtained in controls and in amnesic patients refute the existence of CIPC for forgotten choices, and show that episodic memory is required for CIPC to occur.

## Discussion

Taken together, our results show that, contrarily to what has been reported before the discovery of the regression to the mean artifact, amnesic patients do no show CIPC. Moreover, CIPC is observed both in young and older controls as well as in amnesic patients exclusively for remembered items. Accordingly, we discovered a modulation of left hippocampal activity confirmed by two functional brain-imaging methods, immediately before CIPC. The combination of these three lines of evidence suggests a causal implication of hippocampal activity and of conscious episodic memory in CIPC. Rather than being an automatic and unconscious phenomenon, CIPC is rather a high-level complex cognitive process.

Interestingly, our hypothesis may shed light on recent works reporting CIPC effects resistant to the statistical artifact discovered by Chen and Risen. In particular, Johansson and colleagues^23^ used the choice blindness paradigm to manipulate choices unbeknownst to the participants, and reported a spread effect and a significant bias on subsequent choices. As reported by the authors: “This demonstrates that the participants come to prefer the face they were led to believe they liked”. According to our hypothesis these results would rely on the recall into episodic memory of the manipulated information. This prediction could be tested in future experiments. In accordance with our conception of CIPC as a ‘high-level’ cognitive process permeable to conscious top-down influences, Luo and Yu recently used the same choice blindness paradigm to study the impact of actual choice and perceived choice, and showed that CIPC could be modulated by explicit manipulation^24^. Sharot *et al*.^5^ used a RCR/RRC design to probe very long-lasting effects (2.5 years). They found a significantly higher spread for RCR than for RRC, but by definition the choice-rating delays were highly asymmetrical between the RCR condition (both rating-choice delays were short) and the RRC condition (the delay between the first rating and the choice was very long). This asymmetry may raise an issue to interpret spread values. Three other recent studies used an ingenious blind choice condition preventing subjects’ choices from revealing more information about their real preferences^8^,^25^,^26^. However, these results are not immune from criticism. Indeed, the methodology used by Egan *et al*. to test infants and monkeys does not guarantee a univocal interpretation. In infants, attitude change was estimated by a second blind choice, which does not reflect preferences. Moreover, in monkeys, attitude change was assessed by 10 open choices, following the critical blind choice. Note, however, that according to cognitive dissonance theory, these choices should influence each other in turn, so the results are hard to interpret (see refs 27, 28, 29 for detailed reviews of this study). Moreover, Sharot *et al*.’s 2010 study reported a significant spread only for chosen items while the canonical spread was marginal.

In the light of our findings, we propose that a causal relation would exist between episodic memory for previous choices and CIPC during subsequent intentional ratings or decisions. More specifically we speculate that it may reflect a metacognitive process aiming at assuring a homeostatic regulation of conscious coherence between our past remembered actions and our current beliefs, values and behaviors and that it may be compromised in psychiatric and neurological conditions where self-coherence is compromised. Our metacognitive theoretical hypothesis is clearly in opposition to ‘low-level’ accounts according to which CIPC would reflect unconscious and automatic processes independent from conscious top-down effects and episodic memory. Our results rather strengthen ‘high-level’ theories of CIPC. Our metacognitive account belongs to the class of ‘high-cognitive’ theoretical accounts but differs from traditional cognitive dissonance theory. Indeed, whereas cognitive dissonance theory posits, - since the seminal work of Festinger published in 1957^1^ -, that dissonance would arise immediately after choice stage in the FCP^30^, we rather postulate that CIPC would not occur before a new rating/choice has to be made intentionally (R2 stage). Our results are therefore rather in line with self-perception models of CIPC^31^,^32^, and provide an original and detailed mechanism of the way episodic memory of previous intentional actions would give rise to CIPC (see ref. 3 for a detailed theoretical discussion).

According to our hypothesis we would expect executive function and decision making areas to be correlated to CIPC, - in addition to hippocampus -, once the need for coherence would be accessible through episodic memory recall. Indeed, recent functional neuroimaging^10^,^33^,^34^ and cortical stimulation^35^,^36^ studies revealed the contribution of various prefrontal cortices in CIPC. However, our fMRI analysis did not reveal the contribution of such regions when testing for the critical interaction between memory and condition (RCR vs RRC). This negative result may reflect a lack of sensitivity and could be completed by using a time-resolved method such as SEEG in patients implanted within the frontal lobes.

We close by noting that the potential impact of episodic memory in all the other paradigms associated to cognitive dissonance (such as ‘induced compliance’, ‘belief-disconfirmation’, ‘effort justification’ and ‘misattribution’) deserves to be addressed explicitly.

## Methods

### Ethics Statement

All the experiments described in the manuscript have been approved by the Pitié-Salpetriere’s ethical committee (Comité Consultatif de Protection des Personnes participant à une Recherche Biomédicale-Promotion: CPP 13–41 for the fMRI and behavioral experiments & P091111 for the SEEG experiment). All subjects gave their written informed consents and all the different investigations were conducted according to the principles expressed in the Declaration of Helsinki.

### Participants

#### Experiment 1: Behavior and fMRI in controls

Twenty six participants were recruited through posted advertisements for the fMRI experiment. Six participants were eliminated due to a technical problem with the fMRI machine. The reported analyses were therefore based on 20 subjects (12 males; age range: 20–30; mean ± sd = 25.3 ± 3.7). Participants completed a screening form for significant medical conditions and were paid 80 euros to participate in the experiment.

#### Experiment 2: Behavior and intracranial electrophysiological recordings in epileptic patients

Six epileptic patients (3 males, age range: 25–46, mean ± sd = 35.8 ± 7.2) participated in this study. Neuropsychological assessment revealed normal or mildly impaired general cognitive functioning. These patients suffered from drug-refractory focal epilepsy and were implanted stereotactically with depth electrodes as part of a presurgical evaluation. Implantation sites were selected on purely clinical criteria, with no reference to the present protocol and included the left hippocampus region. One of these patients (patient S2135) was also implanted with microelectrodes recording multi-unit activities at the most internal extremity of the left hippocampus electrode.

#### Experiment 3: Behavior in amnesic Mild Cognitive Impairment (aMCI) patients and matched controls

Twelve amnesic patients (5 males, age range: 50–84, mean ± sd = 69.3 ± 10) and 12 matched control participants (5 males, age range: 54–87, mean ± sd = 73.25 ± 10.4) participated in this part of the experiment. Amnesic patients were recruited and tested at the Memory and Alzheimer’s disease institute at the Pitié-Salpêtriere hospital in Paris (IM2A). Controls were recruited through posted advertisements.

### Stimuli and procedure

#### Experiment 1: Behavior and fMRI in controls

##### Overview

The experiment was composed of 5 consecutive blocks, during which fMRI data were acquired: two rating blocks (Rating 1 and 2) and two Choice blocks (Choice 1 and 2) and one repetition detection block. Finally, a memory test and a familiarity assessment were performed after the scanning sessions.

##### Stimuli

Stimuli consisted of 120 colored images of potential vacation destinations. In Rating blocks and in the repetition detection block, one image was presented at the center of the screen with the destination name printed below the image (font size = 30) in each trial. In Choice blocks, two images and their respective names were presented to the left and to the right of the screen, in each trial. The order in which stimuli were presented within each block was random.

##### Rating 1

Subjects viewed 120 destinations and were asked on each trial to report how much they would like to spend their vacation in that given destination on an eight point scale (1 = “I do not want to go there at all”, 8 = “I would love to go there”). Each trial began with a fixation point that lasted 2 to 3 seconds. Then the destination appeared for 3 second. Subsequently, subjects were presented with an image of two schematic hands with a number from 1 to 8 above each finger (except the thumbs) that randomly varied positions from trial to trial. Subjects were instructed to respond with the finger corresponding to the rating they would like to give. This technique allowed us to avoid different handedness related biases. This screen was presented until response. If subjects answered in less than 3 seconds, a fixation dot was presented, so the interval between the end of the destination presentation and the next trial lasted at least 3 seconds.

##### Choice 1

Following the Rating1 block, subjects were presented with pairs of destinations they had rated equally (difference of R1 scores ≤ 1) and had to indicate with a button press at which one they would rather take their vacation. Note that all individual medians of R1 scores differences were null. The mean of averaged R1 differences was equal to 0.12 ± 0.04. Importantly, RCR and RRC pairs did not differ in terms of R1 scores difference in each of the groups we tested (fMRI controls; SEEG patients; MCI controls; MCI patients; all paired t-tests p-values were ≥ 0.1). Similarly no significant difference could be observed between remembered and forgotten pairs, both for RRC and RCR conditions (all p-values ≥ 0.2). In each of the Choice tasks, subjects viewed 25 pairs of destinations based on ratings given in Rating 1. This means a total of 100 destinations out of the 120 were used to form the Choice blocks. The 20 remaining destinations (the same ones for all subjects) were used as repetition targets for the following repetition detection task described below.

Out of the 100 destinations 50 were used to form 25 pairs in Choice 1 and the 50 other formed the 25 pairs of Choice 2. Each trial began with a fixation point that lasted 2 to 3 seconds. Then the paired destinations appeared for 3 seconds, followed by a fixation point, during which subjects had to give their choice. This fixation screen lasted at least 3 seconds and at most until subject’s response.

##### Incidental task: Repetition detection task

In this task subjects were presented with all 120 destinations as each appeared at the screen with the same presentation time as rating 1 and rating 2. 1/6th of the time either the destination name or the image, or both were repeated contiguously (only for the 20 images mentioned in Choice 1 description). Subjects had to press a button to signal that they detected this repetition. This repetition detection task was implemented in order to make sure subjects were attentively attending all destinations and their respective names. The main aim of this block was to probe the timing of preference change during a passive condition. Analyses of this block did not reveal any significant result. Given the focus of the present study on memory effects, we do not report here these analyses for sake of concision.

##### Rating 2

This block was strictly identical to Rating 1.

##### Choice 2

This block was strictly identical to Choice 1, but only included the other half of the stimuli as described earlier.

##### Post-scanning questions

After the scanning sessions, participants were asked to perform two additional tests outside the fMRI. First they performed a memory test concerning the choices they had made in Choice 1 and Choice 2 sessions. In order to avoid any explicit memorization of the items, subjects were not informed about this memory test at the beginning of the experiment. Every trial began with the appearance of a destination picture and its name at the center of the screen. Below the item, were stated the options “chosen” and “rejected” at the left and right of the screen. Using the keyboard arrows, subjects had to indicate whether they remember choosing or rejecting that given item during the choice tasks. This question tested objective memory of the choices. After giving their answer, appeared a sentence at the center of the screen “Are you sure of your response?” underneath which were presented the two options “ I am sure” and “I guessed” at left and right of the screen. Again subjects had to use the keyboard arrow to specify their answer. This second question tested the more subjective aspect of subjects’ memory. There was no time limitation and the trials concerned all 100 items that were seen during Choice 1 and Choice 2.

Finally, subjects rated the familiarity of each of the 120 destinations using an eight-point scale on the pc’s numeric keypad (1 = “I do not know this country at all”, 8 = “I am very familiar with this country”).

#### Experiment 2: Behavior and intracranial electrophysiological recordings in epileptic patients

The procedure of this experiment was strictly identical to the fMRI experiment in all regards expect in the three following aspects:

1. Given the gain in temporal resolution of SEEG, as compared to fMRI, pictures of the destinations were presented for a duration of 2 seconds rather than 3 seconds. This shortened the overall length of the experiment making it more adapted for implanted patients.
2. As opposed to the fMRI experiment, in which handedness related activations were important to control, in rating 1 and rating 2 of this experiment subjects did not respond using a schematic hand but rather the PC’s numeric keypad.
3. For sake of concision with these patients, the experiment did not include the familiarity test.

#### Experiment 3: Behavior in amnesic Mild Cognitive Impairment (aMCI) patients and matched controls

The procedure of this experiment was strictly identical to the fMRI experiment in all regards expect in the three following aspects:

1. There were neither incidental task nor familiarity test in this experiment.
2. As opposed to the fMRI experiment in which handedness related activations were important to control, in rating 1 and rating 2 of this experiment subjects did not respond using a schematic hand but rather the PC’s numeric keypad.
3. Subjects had to perform 30 rather that 25 choices in each of the choice blocks.

## Additional Information

**How to cite this article:** Chammat, M. *et al*. Cognitive dissonance resolution depends on episodic memory. *Sci. Rep.* **7**, 41320; doi: 10.1038/srep41320 (2017).

**Publisher's note:** Springer Nature remains neutral with regard to jurisdictional claims in published maps and institutional affiliations.

## Supplementary Material

Supplementary Information
