# Supplementary material for: Cognitive dissonance resolution depends on episodic memory
Source: Sci Rep. 2017 Jan 23;7:41320. doi: 10.1038/srep41320 (PMC5256105; doi:10.1038/srep41320)
Supplement: Supplementary Information [file srep41320-s1.doc]

**Supplementary Information**

**Title:**

**Cognitive dissonance resolution depends on episodic memory**

**Authors**

Mariam Chammat^1,2^, Imen El Karoui^1,2^, Sébastien Allali^1,2^, Joshua Hagège^1,2^, Katia Lehongre^1,2,3^, Dominique Hasboun^1,2,4^, Michel Baulac^1,2,4,5^, Stéphane Epelbaum^1,2,5^, Agnès Michon^5^, Bruno Dubois^1,2,4,5^, Vincent Navarro^1,2,4,5,6^, Moti Salti^7^, Lionel Naccache^1,2,4,5,6^*

Corresponding author: [lionel.naccache@upmc.fr](mailto:lionel.naccache@upmc.fr)

1. INSERM, U 1127, F-75013, Paris, France

2. Institut du Cerveau et de la Moelle épinière, ICM, PICNIC Lab, F-75013, Paris, France

3. CENIR, Centre de NeuroImagerie de Recherche, Paris, France

4. Sorbonne Universités, UPMC Univ Paris 06, Faculté de Médecine Pitié-Salpêtrière, Paris, France

5. AP-HP, Groupe hospitalier Pitié-Salpêtrière, Department of Neurology, Paris, France

6. AP-HP, Groupe hospitalier Pitié-Salpêtrière, Department of Neurophysiology, Paris, France

7. Ben-Gurion University, Department of Brain and Cognitive Science, Beer-Sheva, Israel

**Methods**

**Behavioral analysis**

In all three experiments subjects’ responses during both ratings were extracted using Matlab 2013a (Mathworks). A linear mixed model was used to analyze the relationship between memory and spread in this task. Indeed, as opposed to a classical ANOVA, such a model does not require prior averaging of the spread for each subject and thus offers the possibility to handle the heteroskedasticity related to the unbalanced number of items in each condition. Significance of the fixed effects was assessed using the Kenward-Roger approximation for degrees of freedom of the denominator, with the ‘lmerTest’ package in R. Restricted analyses were performed using t-tests.

In order to perform single-subject analyses of behavioral data in each of the epileptic patients, we computed the critical interaction value between memory and condition and then compared it to the distribution observed in the population of healthy subjects (N=30 corresponding to 20 fMRI controls + 10 pilot controls) using a statistical methodology based on non-central t-distributions developed by Crawford and Garthwaite .

**Functional MRI Data Acquisition and Analysis**

Functional imaging was conducted using a 3 Tesla Siemens Verio scanner and a multiband echo-planar imaging sequence sensitive to brain oxygen-level-dependent (BOLD) contrast to produce 45 continuous 2.5-mm thick transaxial slices covering nearly the entire cerebrum (repetition time = 1.022 ms; echo time = 25 ms; flip angle = 60°; field of view = 100 mm2 view = 100 mm2 80 × 80 matrix; voxel dimensions = 2.5 × 2.5 ×2.5 mm). A high-resolution anatomical T1-weighted image was also acquired for each subject for anatomical localization.

fMRI data were analyzed using SPM8 (SPM8, Wellcome Department of Cognitive Neurology, London, United Kingdom) toolbox. Functional images were realigned, unwarped using the FSL "Topup" toolbox in order to correct EPI distortions due to B0 field inhomogeneity (Andersson et al., 2003) and then normalized into Montreal Neurological Institute standard stereotactic space. The normalized fMRI data were spatially smoothed with a Gaussian kernel of 8 mm (full-width at half-maximum) in the x, y, and z axes. We discarded the first four volumes to allow for stabilization of the magnetization before data analysis.

Two main analyses were performed. GLM 1 aimed at identifying brain regions that were positively correlated with subjects' reported preference for each destination. This model was estimated using data from Rating 1 only so that any changes in self-report preferences from the first to second Preference tasks remain independent from changes in striatal activity as proposed by Izuma et al. (2010). Rating values from all 120 trials of rating 1 were used as a covariate. This model included 3 regressors: (i) each vacation destination stimulus onset (ii) stimulus onset modulated by subjects' reported preference for each destination and (iii) stimulus onset modulated by reported familiarity for each destination.

GLM 2 was designed to examine the neural correlates of our behavioral finding showing a significant spread difference between the RCR and RRC conditions only for memorized items. To this aim, we compared brain activity for memorized versus forgotten items in the RCR compared to RRC conditions. Trials from Rating 2 were classified into 4 conditions: (i) Remembered items in the RCR condition, (ii) Forgotten items in the RCR condition, (iii) Remembered item in the RRC condition, (iv) Forgotten items in the RRC condition. The anatomical region of interest (ROI), including bilateral hippocampi was defined using the SPM WFU PickAtlas tool (Maldjian et al., 2003, 2004).

**SEEG Data Acquisition and Analysis**

**Electrode implantation and localization**

Patients were implanted intracerebrally with depth electrodes, each bearing four to ten recording sites (Ad-TechMedical Instruments, Racine, WI, US). To compare recording sites position and summarize brain activations across patients, their coordinates were obtained after normalizing the anatomical three-dimensional post-implantation MRI onto the template from the Montreal Neurological Institute (MNI), using SPM8 software (http://www.fil.ion.ucl.ac.uk/spm).

**Data acquisition and preprocessing**

Data were acquired with an audio-video-EEG monitoring system (Neuralynx for 5 patients and Micromed for one patient), which allowed for simultaneous acquisition of data from up to 128 EEG channels sampled at 4000Hz (Neuralynx) and 1024 Hz (Micromed) respectively. Data were analyzed with Fieldtrip toolbox (Oostenveld et al. 2011) and Matlab 2011a (The Mathworks, Inc., Natick, MA, USA). SEEG signal was subsampled at 250Hz. All the analyses were done at the electrode level, as the position of the recording sites differed from one patient to another. Epochs were extracted from -300ms to 1000ms relative to the onset of items during rating 2 session (R2). To avoid artifacts, recording sites exceeding the threshold of 300 µV in more than 5% of the epochs were excluded. All signals were re-referenced to their nearest neighbor on the same electrode (bipolar montage). In the following, we will refer to these bipolar montages as ‘electrodes’. All data were visually inspected to discard any trial with epileptic activity. The signal was band-pass filtered off-line from 0.5 to 20 Hz using a fourth-order Butterworth filter in forward and reverse directions in order to avoid phase-shift and a baseline correction was applied, by subtracting the mean voltage in the [-300ms to 0ms] window.

**Electrode selection**

We analyzed SEEG signal of bipolar montages of adjacent recording sites for which the closest site was located less than 30mm away from the left hippocampus (MNI Coordinates : -14 -24 -12) peak of fMRI activation reported in controls for the interaction between RCR/RRC and episodic memory of the choice. The number of bipolar montages within or in the close vicinity of left hippocampus was respectively equal to 11 (patient 1), 7 (patient 2) and 5 (patient 3).

**Statistical analyses of SEEG signal**

We computed the interaction between condition (RCR/RRC) and memory (remembered/forgotten) during rating 2 session. To do so we first ran an independent t-test for each time sample and for each bipolar montage ([RCR remembered and RRC forgotten trials] versus [RCR forgotten and RRC remembered trials]) on the [200ms to 600ms] temporal window after item onset, traditionally associated with recall in episodic memory. For each bipolar montage, we only considered effects reaching a threshold of p-value≤0.05 for a minimum of ten consecutive samples (40ms). For each temporal cluster we then computed the sum of absolute values of t-value across the significant successive samples.

In order to control for type I errors generated by multiple comparisons across time, we used a nonparametric Monte Carlo procedure previously described and used . We computed N= 1000 permutations by shuffling trial labels. For each permutation, we repeated the t-test procedure described for real data. We identified temporal clusters satisfying our criterion (p≤0.05 for a minimum of ten consecutive samples). Then for each permutation we kept the maximal value of t-sum. At the end of this procedure we could then assess the significance of each effect observed in real data by computing the probability to observe it within the distribution of shuffled datasets . We only considered effects with an observed probability inferior or equal to 0.05. Finally, in order to correct for multiple comparison over electrodes, we used a False Discovery Rate (FDR) correction (FDR=0.2) on p-value obtained at the electrode level with the Monte Carlo procedure .

**Statistical analysis of the relation between behavior and SEEG signal**

In order to test the relation between the behavioral and the SEEG interaction values across the 6 implanted patients, we calculated the corresponding contingency table, and then computed the one-sided Bayesian factor for H1 (BF_10_) using JASP software (Team, J., Version 0.8.0.0, 2016.) and interpreted it using Jeffrey’s terminology.

**Supplementary Tables**

**Table S1: Areas correlated with items ratings during the first rating**

The brain valuation system’s activity correlated with ratings (statistical threshold was set at p < 0.001 for height (uncorrected) and p < 0.05 Family wise error (FWE) cluster correction).

**Table S2: Epileptic patients characteristics**

**Table S3: Characteristics of the amnestic mild-cognitive impairments (aMCI) patients**

Neuropsychological tests included the Mini-Mental State Evaluation (MMSE), the Frontal Assessment Battery and the Grober and Buschke test of verbal episodic memory. aMCI patients were matched to controls in terms of education level. In France, CPS (Certificate of Primary Studies) refers to the end of Primary school and BAC (Baccalaureate) refers to the end of high school.

**Table S4: Characteristics of matched controls of the aMCI patients**

Neuropsychological tests included the Mini-Mental State Evaluation (MMSE), the Frontal Assessment Battery and the Grober and Buschke test of verbal episodic memory. Controls were matched to aMCI patients in terms of education level. In France, CPS (Certificate of Primary Studies) refers to the end of Primary school and BAC(Baccalaureate) refers to the end of secondary school.

1. Crawford, J.R. and P.H. Garthwaite, *Investigation of the single case in neuropsychology: confidence limits on the abnormality of test scores and test score differences.* Neuropsychologia, 2002. **40**(8): p. 1196-208.

2. Tibon, R. and D.A. Levy, *The time course of episodic associative retrieval: electrophysiological correlates of cued recall of unimodal and crossmodal pair-associate learning.* Cognitive, affective & behavioral neuroscience, 2014. **14**(1): p. 220-35.

3. Gaillard, R., S. Dehaene, C. Adam, S. Clemenceau, D. Hasboun, M. Baulac, L. Cohen and L. Naccache, *Converging intracranial markers of conscious access.* PLoS Biol, 2009. **7**: p. e61.

4. Naccache, L., R. Gaillard, C. Adam, D. Hasboun, S. Clemenceau, M. Baulac, S. Dehaene and L. Cohen, *A direct intracranial record of emotions evoked by subliminal words.* Proc Natl Acad Sci U S A, 2005. **102**(21): p. 7713-7717.

5. Nichols, T.E. and A.P. Holmes, *Nonparametric permutation tests for functional neuroimaging: a primer with examples.* Human brain mapping, 2002. **15**(1): p. 1-25.

6. Benjamini, Y. and Y. Hochberg, *Controlling the False Discovery Rate: a Practical and Powerful Approach to Multiple Testing.* J. R. Statist. Soc., 1995. **57**: p. 289-300.

7. Gunel, E. and J. Dickey, *Bayes factors for independence in contingency tables.* Biometrika, 1974. **61**(3): p. 545-557.

8. Jeffreys, H., *Theory of Probability*. Classic Texts in the Physical Sciences1961, Oxford: Oxford Univ. Press.
